# Brain abscesses in an immunocompromised patient with a soft tissue mass

**DOI:** 10.4102/sajid.v39i1.669

**Published:** 2024-11-29

**Authors:** Thokozani Mwase, Phineas Mapiye, Boitumelo Mashigo, Camilla le Roux, Tamsin Lovelock

**Affiliations:** 1 Department of Internal Medicine, Faculty of Medicine and Health Sciences, Stellenbosch University, Cape Town, South Africa; 2 Department of Medical Imaging and Clinical Oncology, Faculty of Medicine and Health Sciences, Stellenbosch University, Cape Town, South Africa; 3 Department of Infectious Diseases, Faculty of Medicine and Health Sciences, Stellenbosch University, Cape Town, South Africa

**Keywords:** *Nocardia*, brain abscess, immunocompromised, HIV, CNS, nocardiosis

## Abstract

Nocardiosis is a rare opportunistic infection and may be misdiagnosed as tuberculosis in the immunocompromised patient. This case report highlights the importance of doing tissue cultures in immunocompromised individuals to correctly identify *Nocardia* spp. and initiate appropriate treatment timeously.

**Contribution:**

This case report describes a typical case of disseminated nocardiosis with brain abscesses in an immunocompromised patient who would have typically been treated as disseminated tuberculosis.

## Introduction

*Nocardia farcinica* was the first described in 1989 by Trevisan.^1^ *Nocardia* spp. are gram-positive, aerobic and weakly acid-fast filamentous actinomycetes bacteria which are found globally in soil and decaying plant material.^2^ Human infection arises because of inhalation or direct inoculation of the organism. In immunocompetent hosts, *Nocardia* spp. can cause primary cutaneous nocardiosis, but may also manifest as pulmonary and disseminated infections in immunocompromised hosts, with or without soft tissue involvement. As many as 30% of patients with pulmonary disease will develop central nervous system (CNS) involvement in the form of brain abscesses.^3^ Risk factors for invasive *Nocardia* infections include chronic glucocorticoid therapy, malignancy, organ transplantation, HIV infection, diabetes mellitus, structural lung disease and chronic granulomatous disease.^4^ *Nocardia* spp. may require prolonged incubation in culture or may be missed with standard laboratory techniques. The delay in initiating appropriate therapy influences the prognosis of patients with disseminated nocardiosis.

Infection with *Nocardia* may be challenging to differentiate from pulmonary or disseminated tuberculosis (TB). This is not usually a concern for patients in whom a diagnosis of TB has been confirmed by molecular diagnostics or culture, but it may be more significant for patients initiated on TB treatment empirically, or purely based on a suggestive clinical picture. *Nocardia* infection should be considered as a potential alternative diagnosis in patients with respiratory and constitutional symptoms, with or without CNS involvement, in whom TB work-up is negative, or who have demonstrated a poor clinical response to empiric TB treatment.

## Case presentation

**FIGURE 1:**
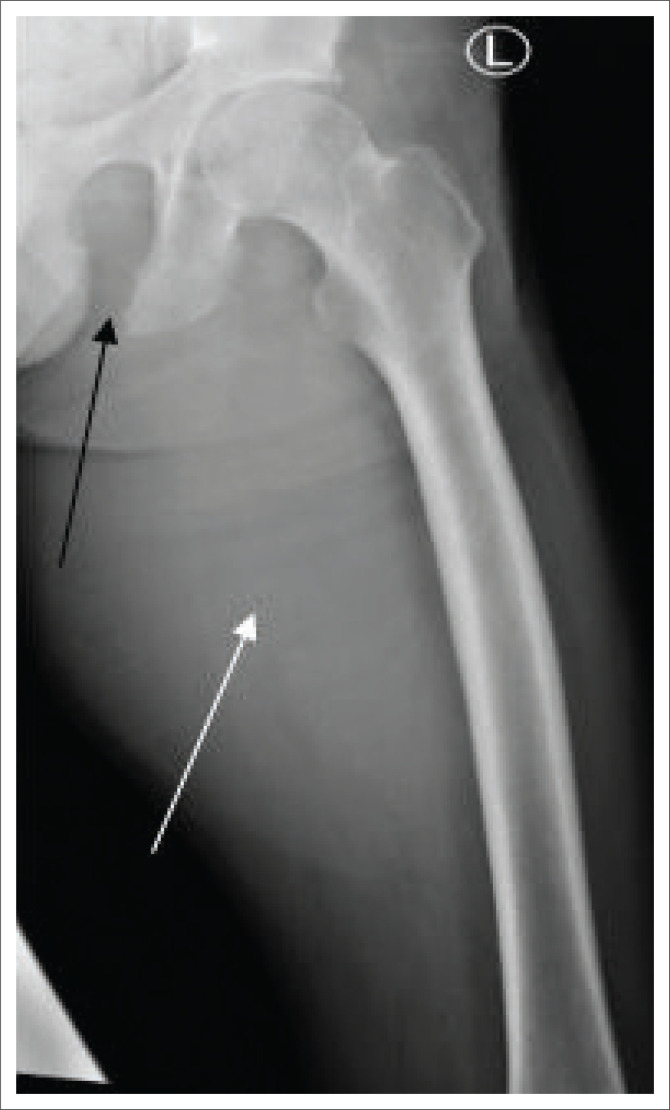
Left frontal thigh radiograph. Left thigh prominent soft tissue swelling (white arrow) associated with erosion of the left inferior pubic ramus (black arrow).

A 33-year-old male presented to an academic hospital in the Western Cape province of South Africa in July 2023 with new onset confusion, a productive cough, weight loss and night sweats. The social background of the patient includes that he is unemployed with poor living circumstances. The patient had been diagnosed with HIV with a nadir CD4 count of 1 cells/uL in 2020 and had interrupted antiretroviral therapy for 6 months. He previously had drug-sensitive pulmonary TB in 2022, for which he had completed 6 months of treatment. He initially presented in 2019 with soft tissue abscesses involving the left thigh, which were drained, and a radiograph was done, which showed an erosion of the left ischial tuberosity and inferior pubic rami (Figure 1). He was treated with oral co-amoxiclav for 5 days. No microscopy and culture specimens were requested during this admission. In June 2023, 1 month prior to this admission, he had presented with soft tissue swelling in his left axilla and left thigh to the surgical department. An incision and drainage of the thigh and axilla abscess were performed, and he received a 5-day course of oral co-amoxiclav again. Specimens were sent for microscopy and culture, and the patient was advised to visit his day hospital to review the results; however, he was unfortunately lost to follow-up after discharge from the hospital.

Clinically he was cachectic, tachycardic and pyrexial with a heart rate of 130 beats per minute (bpm), blood pressure of 100/55 mmHg and temperature of 38.1 °C. He was confused and not oriented to time, place and person. No motor or sensory deficits were detected on examination, and there were no signs of cerebellar impairment. The respiratory examination was notable for features of consolidation on left upper lung zone. He had scars in the left axilla and his left thigh from a previous incision and drainage performed in June 2023.

The patient was admitted to internal medicine and further investigations were requested. Blood results were remarkable for hyponatremia of 125 mmol/L, leucocytosis of 12.11 × 10^9^/L, microcytic anaemia with a haemoglobin of 10.4 g/dL and mean cell volume of 76 fL. Review of his previous laboratory investigations revealed that *N. farcinica* had been isolated from the aspirate sent during his previous admission in June 2023; unfortunately, the susceptibilities could not be done as the sample became contaminated. Additional investigations are summarised in Table 1.

**TABLE 1:** Microbiological laboratory investigations.

Date done	Investigation	Result
10 June 2023	Gram stain Pus aspirate from axilla and thigh microscopy PCR and DNA sequencing of 16S rRNA Pus aspirate GeneXpert MTB/Rif Ultra	Branching gram-positive bacilli *Nocardia* spp. *Nocardia farcinica* MTB not detected
10 July 2023	Urinary LAM Toxoplasma serology	Positive IgG-positive, IgM-negative
14 July 2023	Blood mycobacterial culture	Negative
14 July 2023	Sputum GeneXpert	Negative

LAM, lipoarabinomannan; PCR, polymerase chain reaction; MTB, mycobacterium tuberculosis; rRNA, ribosomal ribonucleic acid; DNA, deoxyribonucleic acid; Rif, rifampicin; IgG, immonuglobulin G, IgM, immunoglobulin M.

**FIGURE 2:**
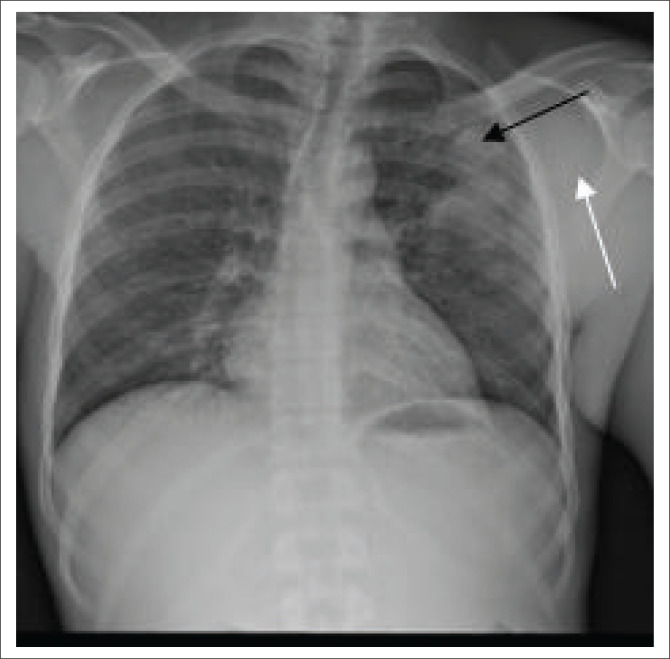
Frontal chest radiograph: left upper lung airspace opacity with internal cavitation (black arrow) and left axillary soft tissue swelling (white arrow).

**FIGURE 3:**
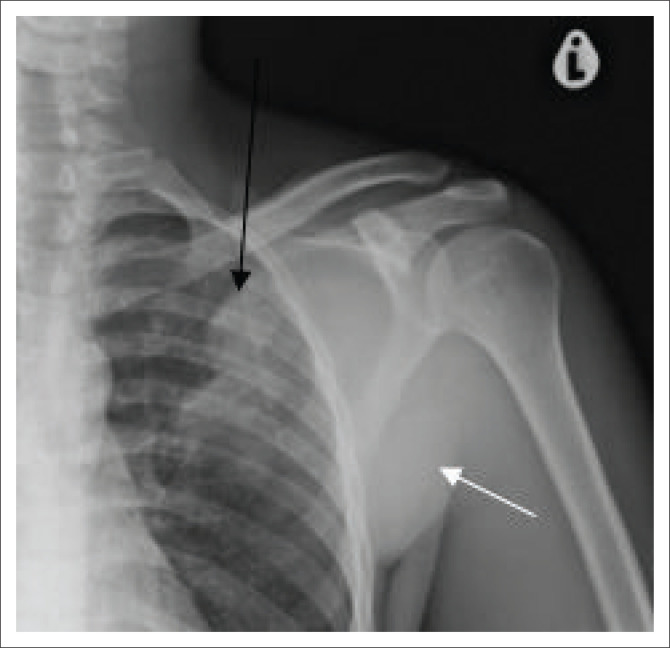
Frontal radiograph of the left shoulder: Left axillary soft tissue prominence (white arrow). Left upper lobe air space disease, with early internal cavitation (black arrow).

**FIGURE 4:**
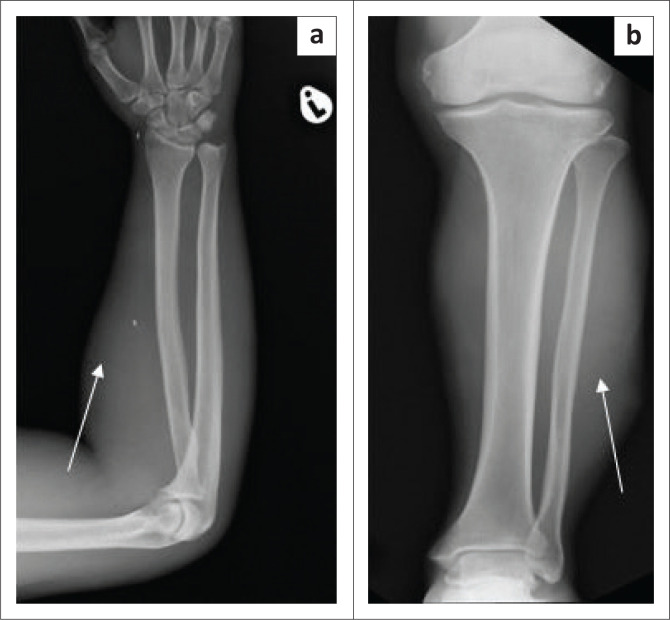
(a) Left distal forearm lateral soft tissue swelling (white arrow). No bone erosion. Small wrist and forearm radio-densities are unchanged and likely related to prior trauma. (b) Frontal projection of the left lower limb demonstrated lateral soft tissue swelling (white arrow). No fibular erosion.

**FIGURE 5:**
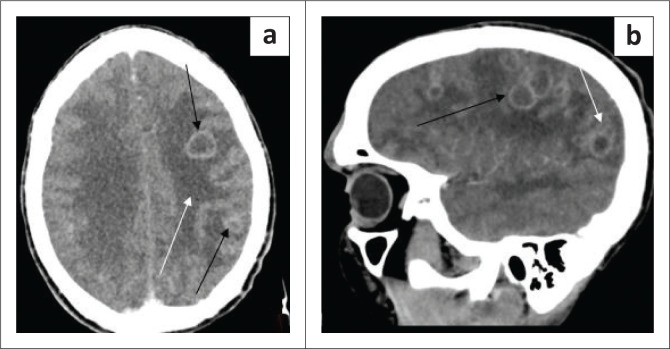
(a) Axial post-contrast computed tomography (CT) brain demonstrates vasogenic oedema (white arrow) and rim-enhancing lesions in the left parietal lobe (black arrows). (b) Sagittal post-contrast brain CT demonstrated loculated rim-enhancing lesions (black arrow), of which some are bilobed (white arrow).

The chest radiograph (Figure 2) demonstrated left upper lobe air space consolidation with cavitation and left axillary soft tissue swelling (Figure 2 and Figure 3). Radiographs of the Left distal forearm (Figure 4a) and lower limb (Figure 4b) demonstrated soft tissue swellings with no bony erosions. Computed tomography (CT) imaging of the head was performed, which demonstrated multiple supra- and infra-tentorial abscesses (Figure 5a and 5b), with a bilobed, multi-loculated morphology with marked mass effect.

The patient was initiated on oral trimethoprim-sulfamethoxazole 320 mg/1600 mg 12 hourly, imipenem 500 mg intravenous (IV) 6 hourly and amikacin 750 mg IV daily which he received for 6 weeks (doses were calculated according to the patient’s weight, and trimethoprim-sulfamethoxazole was not available as an intravenous preparation at the time, so was given by the oral route). There was cerebral oedema with mass effect on CT scan as shown in Figure 5, hence intravenous dexamethasone at 8 mg 8 hourly was also administered. The toxoplasma serology revealed that the IgG was positive with high avidity of > 60% which was supporting previous exposure of toxoplasmosis, but reactivation could not be ruled out. He had a good clinical response to treatment with full recovery of his level of consciousness. The patient was discharged from hospital with a long-term plan to complete 1 year of trimethoprim-sulfamethoxazole. He was re-initiated on antiretrovirals after 4 weeks of nocardiosis treatment.

## Discussion

This case report demonstrates that multiple cutaneous abscesses with poor response to broad-spectrum antibiotics should alert the treating clinician to consider nocardiosis, especially in the presence of severe immunosuppression and when presenting in combination with respiratory and constitutional symptoms. Nocardial infections are broadly classified as pulmonary, CNS, cutaneous or disseminated nocardiosis depending on the location and extent of involvement, though no site in the body is exempt.^5^ Disseminated nocardiosis is defined as the involvement of two non-contiguous sites.^6^ Pulmonary disease typically manifests as multifocal consolidation, likely because of endobronchial spread.^7^ Central nervous system involvement can cause cerebritis, meningitis and abscess formation. *Nocardia* is only found in 1% of brain abscess cohorts and may be misdiagnosed as another infective aetiology or primary CNS tumours or metastases.^8^ Although *Nocardia* is a rare cause of brain abscesses, and involvement is relatively common in patients with systemic nocardiosis, it was found to occur in 44% of patients with systemic nocardiosis in large analysis of 1050 cases.^9^ Diagnosis of *Nocardia* brain abscesses requires aspiration of abscess material and may not be identified without specialised and prolonged culture techniques. The laboratory should be informed in advance when *Nocardia* is suspected. Work-up for other causes of space-occupying lesions such as tuberculoma, toxoplasmosis, bacteria abscesses and cryptococcomas should always be performed simultaneously.^10^

Nocardiosis of the CNS is associated with high morbidity and mortality. A systematic review based on small numbers illustrated that *Nocardia* brain abscesses are typically multifocal, may have a bilobed appearance and found to preferentially involve the infratentorial region and occipital lobes.^11^ Mortality rates for nocardial brain abscesses are around 20%, even lower at 7% when managed with a combination of both surgical and medical therapies.^12^

For nocardial brain abscesses, treatment with high-dose intravenous antibiotics for 3–6 weeks followed by oral treatment for 12 months is recommended.^13^ Switching to an oral regimen is possible if all the following criteria are met: (1) reliable isolate characterisation, (2) clinical improvement, (3) strain susceptible to at least one drug with good oral bioavailability and penetration into the infected sites and (4) no concern regarding digestive absorption.^14^ Trimethoprim-sulfamethoxazole is the most widely used antibiotic, but it can cause serious adverse reactions such as neutropenia or skin rash that might require cessation of treatment. Furthermore, resistance to trimethoprim-sulfamethoxazole has been described in some *Nocardia* spp., so antibiotic susceptibility testing should be performed on cultured isolates.^15^ In South Africa, it has been shown that there is uniform susceptibility to co-trimoxazole, amikacin and linezolid, with a 48.8% susceptibility rate to imipenem.^16^

Our patient, who was severely immunocompromised, had two presentations 4 years apart with soft tissue swellings. Nocardiosis was not suspected as a differential, and identification of a causative organism was not pursued at the first presentation. On the second presentation, the pus aspirate microscopy showed branching gram-positive bacilli, which is what then alerted the microbiologists to prolong culture for possible *Nocardia*. Our patient had positive urine lipoarabinomannan (LAM) despite having negative TB cultures, which we believe could be because of the possibility of cross-reactivity. This has been shown for sputum LAM where there is possibility of cross-reactivity with oral residing organisms such as actinomycetes and *Nocardia* spp. with specificity as low as 15%.^17^ This may be a factor that delays diagnosis and treatment of nocardiosis because of the similarity in clinical presentation to TB and widespread use of urine LAM in patients with advanced HIV. In the correct clinical context, we recommend requesting a gram stain with all specimens, especially when GeneXpert is negative, to ask for not only TB culture but also for *Nocardia*.

## Conclusion

Our case report demonstrates the need for early diagnosis and aggressive treatment of nocardiosis to avoid significant morbidity or mortality. Nocardial brain abscesses must be considered as a part of the differential diagnosis of intra-axial brain lesions in immunocompromised patients. Screening for CNS involvement should be considered when *Nocardia* is isolated at another anatomical site.
